# Good enough practices in scientific computing

**DOI:** 10.1371/journal.pcbi.1005510

**Published:** 2017-06-22

**Authors:** Greg Wilson, Jennifer Bryan, Karen Cranston, Justin Kitzes, Lex Nederbragt, Tracy K. Teal

**Affiliations:** 1 Software Carpentry Foundation, Austin, Texas, United States of America; 2 RStudio and Department of Statistics, University of British Columbia, Vancouver, British Columbia, Canada; 3 Department of Biology, Duke University, Durham, North Carolina, United States of America; 4 Energy and Resources Group, University of California, Berkeley, Berkeley, California, United States of America; 5 Centre for Ecological and Evolutionary Synthesis, University of Oslo, Oslo, Norway; 6 Data Carpentry, Davis, California, United States of America; Ontario Institute for Cancer Research, CANADA

## Abstract

Computers are now essential in all branches of science, but most researchers are never taught the equivalent of basic lab skills for research computing. As a result, data can get lost, analyses can take much longer than necessary, and researchers are limited in how effectively they can work with software and data. Computing workflows need to follow the same practices as lab projects and notebooks, with organized data, documented steps, and the project structured for reproducibility, but researchers new to computing often don't know where to start. This paper presents a set of good computing practices that every researcher can adopt, regardless of their current level of computational skill. These practices, which encompass data management, programming, collaborating with colleagues, organizing projects, tracking work, and writing manuscripts, are drawn from a wide variety of published sources from our daily lives and from our work with volunteer organizations that have delivered workshops to over 11,000 people since 2010.

## Overview

We present a set of computing tools and techniques that every researcher can and should consider adopting. These recommendations synthesize inspiration from our own work, from the experiences of the thousands of people who have taken part in Software Carpentry and Data Carpentry workshops over the past 6 years, and from a variety of other guides. Our recommendations are aimed specifically at people who are new to research computing.

## Introduction

Three years ago, a group of researchers involved in Software Carpentry and Data Carpentry wrote a paper called "Best Practices for Scientific Computing" [1]. That paper provided recommendations for people who were already doing significant amounts of computation in their research. However, as computing has become an essential part of science for all researchers, there is a larger group of people new to scientific computing, and the question then becomes, "where to start?"

This paper focuses on these first accessible skills and perspectives—the "good enough" practices—for scientific computing: a minimum set of tools and techniques that we believe every researcher can and should consider adopting. It draws inspiration from many sources [2–8], from our personal experience, and from the experiences of the thousands of people who have taken part in Software Carpentry and Data Carpentry workshops over the past 6 years.

Our intended audience is researchers who are working alone or with a handful of collaborators on projects lasting a few days to several months. A practice is included in our list if large numbers of researchers use it and large numbers of people are still using it months after first trying it out. We include the second criterion because there is no point in recommending something that people won't actually adopt.

Many of our recommendations are for the benefit of the collaborator every researcher cares about most: their future self (as the joke goes, yourself from 3 months ago doesn't answer email…). Change is hard, and if researchers don't see those benefits quickly enough to justify the pain, they will almost certainly switch back to their old way of doing things. This rules out many practices, such as code review, that we feel are essential for larger-scale development (Section 6).

We organize our recommendations into the following topics (Box 1):

- Data management: saving both raw and intermediate forms, documenting all steps, creating tidy data amenable to analysis.
- Software: writing, organizing, and sharing scripts and programs used in an analysis.
- Collaboration: making it easy for existing and new collaborators to understand and contribute to a project.
- Project organization: organizing the digital artifacts of a project to ease discovery and understanding.
- Tracking changes: recording how various components of your project change over time.
- Manuscripts: writing manuscripts in a way that leaves an audit trail and minimizes manual merging of conflicts.

**Box 1. Summary of practices**

1. Data management
  - Save the raw data.
  - Ensure that raw data are backed up in more than one location.
  - Create the data you wish to see in the world.
  - Create analysis-friendly data.
  - Record all the steps used to process data.
  - Anticipate the need to use multiple tables, and use a unique identifier for every record.
  - Submit data to a reputable DOI-issuing repository so that others can access and cite it.
2. Software
  - Place a brief explanatory comment at the start of every program.
  - Decompose programs into functions.
  - Be ruthless about eliminating duplication.
  - Always search for well-maintained software libraries that do what you need.
  - Test libraries before relying on them.
  - Give functions and variables meaningful names.
  - Make dependencies and requirements explicit.
  - Do not comment and uncomment sections of code to control a program's behavior.
  - Provide a simple example or test data set.
  - Submit code to a reputable DOI-issuing repository.
3. Collaboration
  - Create an overview of your project.
  - Create a shared "to-do" list for the project.
  - Decide on communication strategies.
  - Make the license explicit.
  - Make the project citable.
4. Project organization
  - Put each project in its own directory, which is named after the project.
  - Put text documents associated with the project in the doc directory.
  - Put raw data and metadata in a data directory and files generated during cleanup and analysis in a results directory.
  - Put project source code in the src directory.
  - Put external scripts or compiled programs in the bin directory.
  - Name all files to reflect their content or function.
5. Keeping track of changes
  - Back up (almost) everything created by a human being as soon as it is created.
  - Keep changes small.
  - Share changes frequently.
  - Create, maintain, and use a checklist for saving and sharing changes to the project.
  - Store each project in a folder that is mirrored off the researcher's working machine.
  - Add a file called CHANGELOG.txt to the project's docs subfolder.
  - Copy the entire project whenever a significant change has been made.
  - Use a version control system.
6. Manuscripts
  - Write manuscripts using online tools with rich formatting, change tracking, and reference management.
  - Write the manuscript in a plain text format that permits version control.

## Data management

Data within a project may need to exist in various forms, ranging from what first arrives to what is actually used for the primary analyses. Our recommendations have 2 main themes. One is to work towards ready-to-analyze data incrementally, documenting both the intermediate data and the process. We also describe the key features of "tidy data", which can be a powerful accelerator for analysis [5, 8].

**Fig 1:**
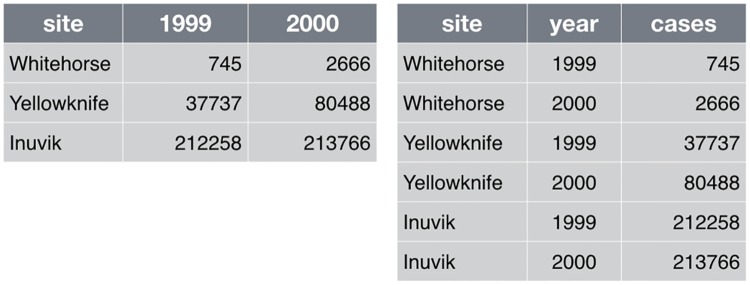
Example of gathering columns to create tidy data.

- **Save the raw data (1a)**. Where possible, save data as originally generated (i.e., by an instrument or from a survey). It is tempting to overwrite raw data files with cleaned-up versions, but faithful retention is essential for rerunning analyses from start to finish, for recovery from analytical mishaps, and for experimenting without fear. Consider changing file permissions to read-only or using spreadsheet protection features so that it is harder to damage raw data by accident or to hand edit it in a moment of weakness.
  Some data will be impractical to manage in this way. For example, you should avoid making local copies of large, stable databases. In that case, record the exact procedure used to obtain the raw data, as well as any other pertinent information, such as an official version number or the date of download.
- **Ensure that raw data are backed up in more than one location (1b)**. If external hard drives are used, store them off-site of the original location. Universities often have their own data-storage solutions, so it is worthwhile to consult with your local Information Technology (IT) group or library. Alternatively, cloud computing resources, like Amazon Simple Storage Service (Amazon S3), Google Cloud Storage, or Microsoft Azure are reasonably priced and reliable. For large data sets, for which storage and transfer can be expensive and time-consuming, you may need to use incremental backup or specialized storage systems, and people in your local IT group or library can often provide advice and assistance on options at your university or organization as well.
- **Create the data you wish to see in the world (1c)**. Create the data set you wish you had received. The goal here is to improve machine and human readability, but not to do vigorous data filtering or add external information. Machine readability allows automatic processing using computer programs, which is important when others want to reuse your data. Specific examples of nondestructive transformations that we recommend at the beginning of analysis include the following:
  *File formats*: Convert data from closed, proprietary formats to open, nonproprietary formats that ensure machine readability across time and computing setups [9]. Good options include CSV for tabular data, JSON, YAML, or XML for nontabular data such as graphs (the node-and-arc kind), and HDF5 for certain kinds of structured data.
  *Variable names*: Replace inscrutable variable names and artificial data codes with self-explaining alternatives, e.g., rename variables called name1 and name2 to personal_name and family_name, recode the treatment variable from 1 vs. 2 to untreated vs. treated, and replace artificial codes for missing data, such as "-99," with NA, a code used in most programming languages to indicate that data are "Not Available" [10].
  *File names*: Store especially useful metadata as part of the file name itself, while keeping the file name regular enough for easy pattern matching. For example, a file name like 2016-05-alaska-b.csv makes it easy for both people and programs to select by year or by location.
- **Create analysis-friendly data (1d)**. Analysis can be much easier if you are working with so-called "tidy" data [5]. Two key principles are as follows:
  *Make each column a variable*: Don't cram 2 variables into one; e.g., "male_treated" should be split into separate variables for sex and treatment status. Store units in their own variable or in metadata, e.g., "3.4" instead of "3.4kg".
  *Make each row an observation*: Data often come in a wide format, because that facilitated data entry or human inspection. Imagine 1 row per field site and then columns for measurements made at each of several time points. Be prepared to gather such columns into a variable of measurements, plus a new variable for time point. Fig 1 presents an example of such a transformation.
- **Record all the steps used to process data (1e)**. Data manipulation is as integral to your analysis as statistical modeling and inference. If you do not document this step thoroughly, it is impossible for you or anyone else to repeat the analysis.
  The best way to do this is to write scripts for *every* stage of data processing. This might feel frustratingly slow, but you will get faster with practice. The immediate payoff will be the ease with which you can redo data preparation when new data arrive. You can also reuse data preparation steps in the future for related projects. For very large data sets, data preparation may also include writing and saving scripts to obtain the data or subsets of the data from remote storage.
  Some data-cleaning tools, such as OpenRefine, provide a graphical user interface but also automatically keep track of each step in the process. When tools like these or scripting are not feasible, it's important to clearly document every manual action (what menu was used, what column was copied and pasted, what link was clicked, etc.). Often, you can at least capture *what* action was taken, if not the complete *why*. For example, choosing a region of interest in an image is inherently interactive, but you can save the region chosen as a set of boundary coordinates.
- **Anticipate the need to use multiple tables, and use a unique identifier for every record (1f)**. Raw data, even if tidy, are not necessarily complete. For example, the primary data table might hold the heart rate for individual subjects at rest and after a physical challenge, identified via a subject ID. Demographic variables, such as subject age and sex, are stored in a second table and will need to be brought in via merging or lookup. This will go more smoothly if subject ID is represented in a common format in both tables, e.g., always as "14025" versus "14,025" in one table and "014025" in another. It is generally wise to give each record or unit a unique, persistent key and to use the same names and codes when variables in 2 data sets refer to the same thing.
- **Submit data to a reputable DOI-issuing repository so that others can access and cite it (1g)**. Your data are as much a product of your research as the papers you write and just as likely to be useful to others (if not more so). Sites such as Figshare, Dryad, and Zenodo allow others to find your work, use it, and cite it; we discuss licensing in Section 3 below. Follow your research community's standards for how to provide metadata. Note that there are 2 types of metadata: metadata about the data set as a whole and metadata about the content within the data set. If the audience is humans, write the metadata (the README file) for humans. If the audience includes automatic metadata harvesters, fill out the formal metadata and write a good README file for the humans [11].

Taken in order, the recommendations above will produce intermediate data files with increasing levels of cleanliness and task specificity. An alternative approach to data management would be to fold all data-management tasks into a monolithic procedure for data analysis, so that intermediate data products are created "on the fly" and stored only in memory, not saved as distinct files.

While the latter approach may be appropriate for projects in which very little data cleaning or processing is needed, we recommend the explicit creation and retention of intermediate products. Saving intermediate files makes it easy to rerun parts of a data analysis pipeline, which in turn makes it less onerous to revisit and improve specific data-processing tasks. Breaking a lengthy workflow into pieces makes it easier to understand, share, describe, and modify. This is particularly true when working with large data sets, for which storage and transfer of the entire data set is not trivial or inexpensive.

## Software

If you or your group are creating tens of thousands of lines of software for use by hundreds of people you have never met, you are doing software engineering. If you're writing a few dozen lines now and again and are probably going to be their only user, you may not be doing engineering, but you can still make things easier on yourself by adopting a few key engineering practices. What's more, adopting these practices will make it easier for people to understand and (re)use your code.

The core realization in these practices is that being *readable*, *reusable*, and *testable* are all side effects of writing *modular* code, i.e., of building programs out of short, single-purpose functions with clearly-defined inputs and outputs [12]. Much has been written on this topic [12–14], and this section focuses on practices that best balance ease of use with benefit for you and collaborators.

- **Place a brief explanatory comment at the start of every program (2a)**, no matter how short it is. That comment should include at least 1 example of how the program is used; remember, a good example is worth a thousand words. Where possible, the comment should also indicate reasonable values for parameters. An example of such a comment is shown below.
  Synthesize image files for testing circularity estimation algorithm.
  Usage: make_images.py -f fuzzing -n flaws -o output -s seed -v -w size
  where:
  -f fuzzing: fuzzing range of blobs (typically 0.0–0.2)
  -n flaws: p(success) for # flaws/sample (e.g. 0.5–0.8)
  -o output: name of output file
  -s seed: random number generator seed (large integer)
  -v: verbose
  -w size: image width/height in pixels (typically 480–800)
  -h: show help message
- **Decompose programs into functions (2b)** that are no more than 1 page (about 60 lines) long. A function is a reusable section of software that can be treated as a black box by the rest of the program. The syntax for creating functions depends on programming language, but generally, you name the function, list its input parameters, and describe what information it produces. Functions should take no more than 5 or 6 input parameters and should not reference outside information.
  The key motivation here is to fit the program into the most limited memory of all: ours. Human short-term memory is famously incapable of holding more than about 7 items at once [15]. If we are to understand what our software is doing, we must break it into chunks that obey this limit, then create programs by combining these chunks. Putting code into functions also makes it easier to test and troubleshoot when things go wrong.
- **Be ruthless about eliminating duplication (2c)**. Write and reuse functions instead of copying and pasting code, and use data structures like lists instead of creating many closely related variables, e.g., create score = (1, 2, 3) rather than score1, score2, and score3.
  Also, look for well-maintained libraries that already do what you're trying to do. All programming languages have libraries that you can import and use in your code. This is code that people have already written and made available for distribution that has a particular function. For instance, there are libraries for statistics, modeling, mapping, and many more. Many languages catalog the libraries in a centralized source, for instance, R has CRAN, Python has PyPI, and so on. Thus, **always search for well-maintained software libraries that do what you need (2d)** before writing new code yourself, but **test libraries before relying on them (2e)**.
- **Give functions and variables meaningful names (2f)**, both to document their purpose and to make the program easier to read. As a rule of thumb, the greater the scope of a variable, the more informative its name should be; while it's acceptable to call the counter variable in a loop i or j, things that are reused often, such as the major data structures in a program, should *not* have 1-letter names. Remember to follow each language's conventions for names, such as net_charge for Python and NetCharge for Java.

**Tab completion**

Almost all modern text editors provide tab completion, so that typing the first part of a variable name and then pressing the tab key inserts the completed name of the variable. Employing this means that meaningful, longer variable names are no harder to type than terse abbreviations.

- **Make dependencies and requirements explicit (2g)**. This is usually done on a per-project rather than per-program basis, i.e., by adding a file called something like requirements.txt to the root directory of the project or by adding a "Getting Started" section to the README file.
- **Do not comment and uncomment sections of code to control a program's behavior (2h)**, since this is error prone and makes it difficult or impossible to automate analyses. Instead, put if/else statements in the program to control what it does.
- **Provide a simple example or test data set (2i)** that users (including yourself) can run to determine whether the program is working and whether it gives a known correct output for a simple known input. Such a "build-and-smoke test" is particularly helpful when supposedly innocent changes are being made to the program or when it has to run on several different machines, e.g., the developer's laptop and the department's cluster.
- **Submit code to a reputable DOI-issuing repository (2j)** upon submission of paper, just as you do with data. Your software is as much a product of your research as your papers and should be as easy for people to credit. DOIs for software are provided by Figshare and Zenodo. Zenodo integrates directly with GitHub.

## Collaboration

You may start working on projects by yourself or with a small group of collaborators you already know, but you should design it to make it easy for new collaborators to join. These collaborators might be new grad students or postdocs in the lab or they might be *you* returning to a project that has been idle for some time. As summarized in [16], you want to make it easy for people to set up a local workspace so that they can contribute, help them find tasks so that they know what to contribute, and make the contribution process clear so that they know how to contribute. You also want to make it easy for people to give you credit for your work.

- **Create an overview of your project (3a)**. Have a short file in the project's home directory that explains the purpose of the project. This file (generally called README, README.txt, or something similar) should contain the project's title, a brief description, up-to-date contact information, and an example or 2 of how to run various cleaning or analysis tasks. It is often the first thing users and collaborators on your project will look at, so make it explicit how you want people to engage with the project. If you are looking for more contributors, make it clear that you welcome contributors and point them to the license (more below) and ways they can help.
  You should also create a CONTRIBUTING file that describes what people need to do in order to get the project going and use or contribute to it, i.e., dependencies that need to be installed, tests that can be run to ensure that software has been installed correctly, and guidelines or checklists that your project adheres to.
- **Create a shared "to-do" list (3b)**. This can be a plain text file called something like notes.txt or todo.txt, or you can use sites such as GitHub or Bitbucket to create a new issue for each to-do item (you can even add labels such as "low hanging fruit" to point newcomers at issues that are good starting points). Whatever you choose, describe the items clearly so that they make sense to newcomers.
- **Decide on communication strategies (3c)**. Make explicit decisions about (and publicize where appropriate) how members of the project will communicate with each other and with externals users/collaborators. This includes the location and technology for email lists, chat channels, voice/video conferencing, documentation, and meeting notes, as well as which of these channels will be public or private.
- **Make the license explicit (3d)**. Have a LICENSE file in the project's home directory that clearly states what license(s) apply to the project's software, data, and manuscripts. Lack of an explicit license does not mean there isn't one; rather, it implies the author is keeping all rights and others are not allowed to reuse or modify the material.
  We recommend Creative Commons licenses for data and text, either CC-0 (the "No Rights Reserved" license) or CC-BY (the "Attribution" license, which permits sharing and reuse but requires people to give appropriate credit to the creators). For software, we recommend a permissive open source license such as the MIT, BSD, or Apache license [17].

**What not to do**

We recommend against the "no commercial use" variations of the Creative Commons licenses because they may impede some forms of reuse. For example, if a researcher in a developing country is being paid by her government to compile a public health report, she will be unable to include your data if the license says "noncommercial". We recommend permissive software licenses rather than the GNU General Public License (GPL) because it is easier to integrate permissively licensed software into other projects; see chapter 3 in [17].

- **Make the project citable (3e)** by including a CITATION file in the project's home directory that describes how to cite this project as a whole and where to find (and how to cite) any data sets, code, figures, and other artifacts that have their own DOIs. The example below shows the CITATION file for the Ecodata Retriever (https://github.com/weecology/retriever); for an example of a more detailed CITATION file, see the one for the khmer project (https://github.com/dib-lab/khmer).

Please cite this work as:

Morris, B.D. and E.P. White. 2013. "The EcoData Retriever: improving access to existing ecological data". PLoS ONE 8:e65848. http://doi.org/doi:10.1371/journal.pone.0065848

## Project organization

Organizing the files that make up a project in a logical and consistent directory structure will help you and others keep track of them. Our recommendations for doing this are drawn primarily from [2, 3].

- **Put each project in its own directory, which is named after the project (4a)**. Like deciding when a chunk of code should be made a function, the ultimate goal of dividing research into distinct projects is to help you and others best understand your work. Some researchers create a separate project for each manuscript they are working on, while others group all research on a common theme, data set, or algorithm into a single project.
  As a rule of thumb, divide work into projects based on the overlap in data and code files. If 2 research efforts share no data or code, they will probably be easiest to manage independently. If they share more than half of their data and code, they are probably best managed together, while if you are building tools that are used in several projects, the common code should probably be in a project of its own. Projects do often require their own organizational model, but below are general recommendations on how you can structure data, code, analysis outputs, and other files. The important concept is that it is useful to organize the project by the types of files and that consistency helps you effectively find and use things later.
- **Put text documents associated with the project in the doc directory (4b)**. This includes files for manuscripts, documentation for source code, and/or an electronic lab notebook recording your experiments. Subdirectories may be created for these different classes of files in large projects.
- **Put raw data and metadata in a data directory and files generated during cleanup and analysis in a results directory (4c)**, where "generated files" includes intermediate results such as cleaned data sets or simulated data, as well as final results such as figures and tables.
  The results directory will usually require additional subdirectories for all but the simplest projects. Intermediate files such as cleaned data, statistical tables, and final publication-ready figures or tables should be separated clearly by file-naming conventions or placed into different subdirectories; those belonging to different papers or other publications should be grouped together. Similarly, the data directory might require subdirectories to organize raw data based on time, method of collection, or other metadata most relevant to your analysis.
- **Put project source code in the src directory (4d)**. src contains all of the code written for the project. This includes programs written in interpreted languages such as R or Python; those written in compiled languages like Fortran, C++, or Java; as well as shell scripts, snippets of SQL used to pull information from databases; and other code needed to regenerate the results.
  This directory may contain 2 conceptually distinct types of files that should be distinguished either by clear file names or by additional subdirectories. The first type is files or groups of files that perform the core analysis of the research, such as data cleaning or statistical analyses. These files can be thought of as the "scientific guts" of the project.
  The second type of file in src is controller or driver scripts that contain all the analysis steps for the entire project from start to finish, with particular parameters and data input/output commands. A controller script for a simple project, for example, may read a raw data table, import and apply several cleanup and analysis functions from the other files in this directory, and create and save a numeric result. For a small project with 1 main output, a single controller script should be placed in the main src directory and distinguished clearly by a name such as "runall". The short example in Box 2 is typical of scripts of this kind; note how it uses 1 variable, TEMP_DIR, to avoid repeating the name of a particular directory 4 times.

**Box 2. Example of a "runall" script**

TEMP_DIR = ./temp_zip_files

echo "Packaging zip files required by analysis tool…"

mkdir $(TEMP_DIR)

./src/make-zip-files.py $(TEMP_DIR) *.dat

echo "Analyzing…"

./bin/sqr_mean_analyze -i $(TEMP_DIR) -b "temp"

echo "Cleaning up…"

rm -rf $(TEMP_DIR)

- **Put compiled programs in the bin directory (4e)**. bin contains executable programs compiled from code in the src directory (the name bin is an old Unix convention and comes from the term "binary"). Projects that do not have any executable programs compiled from code in the src directory will not require bin.

**Scripts versus programs**

We use the term "script" to mean "something that is executed directly as is" and "program" to mean "something that is explicitly compiled before being used". The distinction is more one of degree than kind—libraries written in Python are actually compiled to bytecode as they are loaded, for example—so one other way to think of it is "things that are edited directly" and "things that are not edited directly".

**External scripts**

If src is for human-readable source code and bin is for compiled binaries, where should projects put scripts that are executed directly—particularly ones that are brought in from outside the project? On the one hand, these are written in the same languages as the project-specific scripts in src; on the other, they are executable, like the programs in bin. The answer is that it doesn't matter, as long as each team's projects follow the same rule. As with many of our other recommendations, consistency and predictability are more important than hairsplitting.

- **Name all files to reflect their content or function (4f)**. For example, use names such as bird_count_table.csv, manuscript.md, or sightings_analysis.py. Do not use sequential numbers (e.g., result1.csv, result2.csv) or a location in a final manuscript (e.g., fig_3_a.png), since those numbers will almost certainly change as the project evolves.

The structure shown in Box 3 is a concrete example of how a simple project might be organized following these recommendations. The root directory contains a README file that provides an overview of the project as a whole, a CITATION file that explains how to reference it, and a LICENSE file that states the licensing. The data directory contains a single CSV file with tabular data on bird counts (machine-readable metadata could also be included here). The src directory contains sightings_analysis.py, a Python file containing functions to summarize the tabular data, and a controller script runall.py that loads the data table, applies functions imported from sightings_analysis.py, and saves a table of summarized results in the results directory.

**Box 3. Project layout**

.

|-- CITATION

|-- README

|-- LICENSE

|-- requirements.txt

|-- data

|  |-- birds_count_table.csv

|-- doc

|  |-- notebook.md

|  |-- manuscript.md

|  |-- changelog.txt

|-- results

|  |-- summarized_results.csv

|-- src

|  |-- sightings_analysis.py

|  |-- runall.py

This project doesn't have a bin directory because it does not rely on any compiled software. The doc directory contains 2 text files written in Markdown, 1 containing a running lab notebook describing various ideas for the project and how these were implemented, and the other containing a running draft of a manuscript describing the project findings.

## Keeping track of changes

Keeping track of changes that you or your collaborators make to data and software is a critical part of research. Being able to reference or retrieve a specific version of the entire project aids in reproducibility for you leading up to publication, when responding to reviewer comments, and when providing supporting information for reviewers, editors, and readers.

We believe that the best tools for tracking changes are the version control systems that are used in software development, such as Git, Mercurial, and Subversion. They keep track of what was changed in a file, when, and by whom and synchronize changes to a central server so that multiple contributors can manage changes to the same set of files.

While these version control tools make tracking changes easier, they can have a steep learning curve. Thus, we provide 2 sets of recommendations, (1) a systematic manual approach for managing changes and (2) version control in its full glory, and you can use the first while working towards the second or just jump into version control.

Whatever system you chose, we recommend that you do the following:

- **Back up (almost) everything created by a human being as soon as it is created (5a)**. This includes scripts and programs of all kinds, software packages that your project depends on, and documentation. A few exceptions to this rule are discussed below.
- **Keep changes small (5b)**. Each change should not be so large as to make the change tracking irrelevant. For example, a single change such as "Revise script file" that adds or changes several hundred lines is likely too large, as it will not allow changes to different components of an analysis to be investigated separately. Similarly, changes should not be broken up into pieces that are too small. As a rule of thumb, a good size for a single change is a group of edits that you could imagine wanting to undo in one step at some point in the future.
- **Share changes frequently (5c)**. Everyone working on the project should share and incorporate changes from others on a regular basis. Do not allow individual investigator's versions of the project repository to drift apart, as the effort required to merge differences goes up faster than the size of the difference. This is particularly important for the manual versioning procedure described below, which does not provide any assistance for merging simultaneous, possibly conflicting changes.
- **Create, maintain, and use a checklist for saving and sharing changes to the project (5d)**. The list should include writing log messages that clearly explain any changes, the size and content of individual changes, style guidelines for code and updating to-do lists, and bans on committing half-done work or broken code. See [18] for more on the proven value of checklists.
- **Store each project in a folder that is mirrored off the researcher's working machine (5e)** using a system such as Dropbox or a remote version control repository such as GitHub. Synchronize that folder at least daily. It may take a few minutes, but that time is repaid the moment a laptop is stolen or its hard drive fails.

### Manual versioning

Our first suggested approach, in which everything is done by hand, has 2 additional parts:

- **Add a file called CHANGELOG.txt to the project's docs subfolder (5f)**, and make dated notes about changes to the project in this file in reverse chronological order (i.e., most recent first). This file is the equivalent of a lab notebook and should contain entries like those shown below.

## 2016-04-08

* Switched to cubic interpolation as default.

* Moved question about family's TB history to end of questionnaire.

## 2016-04-06

* Added option for cubic interpolation.

* Removed question about staph exposure (can be inferred from blood test results).

- **Copy the entire project whenever a significant change has been made (5g)** (i.e., one that materially affects the results), and store that copy in a subfolder whose name reflects the date in the area that's being synchronized. This approach results in projects being organized as shown below:

.

|-- project_name

|  |-- current

|  |  |-- …project content as described earlier…

|  |-- 2016-03-01

|  |  |-- …content of 'current' on Mar 1, 2016

|  |-- 2016-02-19

|  |  |-- …content of 'current' on Feb 19, 2016

Here, the project_name folder is mapped to external storage (such as Dropbox), current is where work is being done, and other folders within project_name are old versions.

**Data are cheap, time is expensive**

Copying everything like this may seem wasteful, since many files won't have changed, but consider: a terabyte hard drive costs about US$50 retail, which means that 50 Gigabytes costs less than US$5. Provided large data files are kept out of the backed-up area (discussed below), this approach costs less than the time it would take to select files by hand for copying.

This manual procedure satisfies the requirements outlined above without needing any new tools. If multiple researchers are working on the same project, though, they will need to coordinate so that only a single person is working on specific files at any time. In particular, they may wish to create 1 change log file per contributor and to merge those files whenever a backup copy is made.

### Version control systems

What the manual process described above requires most is self-discipline. The version control tools that underpin our second approach—the one we use in our own projects—don't just accelerate the manual process, they also automate some steps while enforcing others and thereby require less self-discipline for more reliable results.

- **Use a version control system (5h) to manage changes to a project**.
- Box 4 briefly explains how version control systems work. It's hard to know what version control tool is most widely used in research today, but the one that's most talked about is undoubtedly Git. This is largely because of GitHub, a popular hosting site that combines the technical infrastructure for collaboration via Git with a modern web interface. GitHub is free for public and open source projects and for users in academia and nonprofits. GitLab is a well-regarded alternative that some prefer, because the GitLab platform itself is free and open source. Bitbucket provides free hosting for both Git and Mercurial repositories but does not have nearly as many scientific users.

**Box 4. How version control systems work**

A version control system stores snapshots of a project's files in a repository. Users modify their working copy of the project, then save changes to the repository when they wish to make a permanent record and/or share their work with colleagues. The version control system automatically records when the change was made and by whom, along with the changes themselves.

Crucially, if several people have edited 1 or more files simultaneously, the version control system will detect any overlapping changes and require conflicts to be resolved before storing the result. Modern version control systems allow repositories to be synchronized with each other, so that no 1 repository becomes a single point of failure. Tool-based version control has several benefits over manual version control:

- Instead of requiring users to make backup copies of the whole project, version control safely stores just enough information to allow old versions of files to be recreated on demand.
- Instead of relying on users to choose sensible names for backup copies, the version control system timestamps all saved changes automatically.
- Instead of requiring users to be disciplined about completing the change log, version control systems prompt them every time a change is saved. They also keep a 100% accurate record of what was *actually* changed as opposed to what the user *thought* they changed, which can be invaluable when problems crop up later.
- Instead of simply copying files to remote storage, version control checks to see whether doing that would overwrite anyone else's work. If so, they facilitate identifying conflict and merging changes.

### What not to put under version control

The benefits of version control systems don't apply equally to all file types. In particular, version control can be more or less rewarding depending on file size and format. First, file comparison in version control systems is optimized for plain text files, such as source code. The ability to see so-called "diffs" is one of the great joys of version control. Unfortunately, while Microsoft Office files (like the .docx files used by Word) or other binary files, e.g., PDFs, can be stored in a version control system, it is not possible to pinpoint specific changes from 1 version to the next. Tabular data (such as CSV files) can be put in version control, but changing the order of the rows or columns will create a big change for the version control system, even if the actual data have not changed.

Second, raw data should not change and therefore should not require version tracking. Keeping intermediate data files and other results under version control is also not necessary if you can regenerate them from raw data and software. However, if data and results are small, we still recommend versioning them for ease of access by collaborators and for comparison across versions.

Third, today's version control systems are not designed to handle megabyte-sized files, never mind gigabytes, so large data or results files should not be included (as a benchmark for "large", the limit for an individual file on GitHub is 100 MB). Some emerging hybrid systems such as Git Large File Storage (LFS) put textual notes under version control while storing the large data in a remote server, but these are not yet mature enough for us to recommend.

**Inadvertent sharing**

Researchers dealing with data subject to legal restrictions that prohibit sharing (such as medical data) should be careful not to put data in public version control systems. Some institutions may provide access to private version control systems, so it is worth checking with your IT department.

Additionally, be sure not to unintentionally place security credentials such as passwords and private keys in a version control system where it may be accessed by others.

## Manuscripts

An old joke says that doing the research is the first 90% of any project; writing up is the other 90%. While writing is rarely addressed in discussions of scientific computing, computing has changed scientific writing just as much as it has changed research.

A common practice in academic writing is for the lead author to send successive versions of a manuscript to coauthors to collect feedback, which is returned as changes to the document, comments on the document, plain text in email, or a mix of all 3. This allows coauthors to use familiar tools but results in a lot of files to keep track of and a lot of tedious manual labor to merge comments to create the next master version.

Instead of an email-based workflow, we recommend mirroring good practices for managing software and data to make writing scalable, collaborative, and reproducible. As with our recommendations for version control in general, we suggest that groups choose 1 of 2 different approaches for managing manuscripts. The goals of both are to do as follows:

- Ensure that text is accessible to yourself and others now and in the future by making a single master document that is available to all coauthors at all times.
- Reduce the chances of work being lost or people overwriting each other's work.
- Make it easy to track and combine contributions from multiple collaborators.
- Avoid duplication and manual entry of information, particularly in constructing bibliographies, tables of contents, and lists.
- Make it easy to regenerate the final published form (e.g., a PDF) and to tell if it is up to date.
- Make it easy to share that final version with collaborators and to submit it to a journal.

**The first rule is…**

The workflow you choose is less important than having all authors agree on the workflow before writing starts. Make sure to also agree on a single method to provide feedback, be it an email thread or mailing list, an issue tracker (like the ones provided by GitHub and Bitbucket), or some sort of shared online to-do list.

### Single master online

Our first alternative will already be familiar to many researchers:

- **Write manuscripts using online tools with rich formatting, change tracking, and reference management (6a)**, such as Google Docs. With the document online, everyone's changes are in one place and hence don't need to be merged manually.

We realize that, in many cases, even this solution is asking too much from collaborators who see no reason to move forward from graphical desktop tools. To satisfy them, the manuscript can be converted to a desktop editor file format (e.g., Microsoft Word .docx or LibreOffice .odt) after major changes, then downloaded and saved in the doc folder. Unfortunately, this means merging some changes and suggestions manually, as existing tools cannot always do this automatically when switching from a desktop file format to text and back (although Pandoc can go a long way).

### Text-based documents under version control

The second approach treats papers exactly like software and has been used by researchers in mathematics, astronomy, physics, and related disciplines for decades:

- **Write the manuscript in a plain text format that permits version control (6b)**, such as LaTeX or Markdown, and then convert them to other formats, such as PDF, as needed using scriptable tools like Pandoc.

Using a version control system provides good support for finding and merging differences resulting from concurrent changes. It also provides a convenient platform for making comments and performing review.

This approach reuses the version control tools and skills used to manage data and software and is a good starting point for fully reproducible research. However, it requires all contributors to understand a much larger set of tools, including Markdown or LaTeX, Make, BiBTeX, and Git/GitHub.

### Why two recommendations for manuscripts?

We initially recommended that researchers should always use plain text in version control to manage manuscripts. However, several members of our community felt strongly that the learning curve associated with this recommendation was a significant barrier to entry. For example, Stephen Turner wrote:

…try to explain the notion of compiling a document to an overworked physician you collaborate with. Oh, but before that, you have to explain the difference between plain text and word processing. And text editors. And Markdown/LaTeX compilers. And BiBTeX. And Git. And GitHub. Etc. Meanwhile, he/she is getting paged from the OR…

…as much as we want to convince ourselves otherwise, when you have to collaborate with those outside the scientific computing bubble, the barrier to collaborating on papers in this framework is simply too high to overcome. Good intentions aside, it always comes down to, "just give me a Word document with tracked changes", or similar.

Similarly, Arjun Raj [19] said in a blog post:

Google Docs excels at easy sharing, collaboration, simultaneous editing, commenting, and reply-to-commenting. Sure, one can approximate these using text-based systems and version control. The question is why anyone would like to…

The goal of reproducible research is to make sure one can reproduce… computational analyses. The goal of version control is to track changes to source code. These are fundamentally distinct goals, and while there is some overlap, version control is merely a tool to help achieve that and comes with so much overhead and baggage that it is often not worth the effort.

Collaborative editing in something like Google Docs does not have all the benefits of text-based formats (notably, being able to store manuscripts in the same place, and in the same way, as other materials). However, it does meet the requirements that we initially outlined. We still recommend *against* using desktop tools like LibreOffice and Microsoft Word with either email or file-sharing services like Dropbox, as workflows based on these do not scale beyond a small number of participants.

### Supplementary materials

Supplementary materials often contain much of the work that went into the project, such as tables and figures or more elaborate descriptions of the algorithms, software, methods, and analyses. In order to make these materials as accessible to others as possible, do not rely solely on the PDF format, since extracting data from PDFs is notoriously hard. Instead, we recommend separating the results that you may expect others to reuse (e.g., data in tables, data behind figures) into separate, text-format files in formats such as CSV, JSON, YAML, XML, or HDF5. We recommend against more innovative formats in deference to an old saying: "What's oldest lasts longest". The same holds for any commands or code you want to include as supplementary material: use the format that most easily enables reuse (source code files, Unix shell scripts, etc.).

## What we left out

We have deliberately left many good tools and practices off our list, including some that we use daily, because they only make sense on top of the core practices described above or because it takes a larger investment before they start to pay off.

- **Branches**. A branch is a "parallel universe" within a version control repository. Developers create branches so that they can make multiple changes to a project independently. They are central to the way that experienced developers use systems like Git, but they add an extra layer of complexity to version control for newcomers. Programmers got along fine in the days of CVS and Subversion without relying heavily on branching, and branching can be adopted without significant disruption after people have mastered a basic edit-commit workflow.
- **Build tools**. Tools like Make were originally developed to recompile pieces of software that had fallen out of date. They are now also used to regenerate data and entire papers; when one or more raw input files change, Make can automatically rerun those parts of the analysis that are affected, regenerate tables and plots, and then regenerate the human-readable PDF that depends on them. However, newcomers can achieve the same behavior by writing shell scripts that rerun everything; these may do unnecessary work, but given the speed of today's machines, that is unimportant for small projects.
- **Unit tests**. A unit test is a small test of 1 particular feature of a piece of software. Projects rely on unit tests to prevent regression, i.e., to ensure that a change to 1 part of the software doesn't break other parts. While unit tests are essential to the health of large libraries and programs, we have found that they usually aren't compelling for solo exploratory work (note, for example, the lack of a test directory in Noble's rules [3]). Rather than advocating something which people are unlikely to adopt, we have left unit testing off this list.
- **Coverage**. Every modern programming language comes with tools to report the coverage of a set of test cases, i.e., the set of lines that are and aren't actually executed when those tests are run. As with unit testing, this only starts to pay off as projects grow larger and is therefore not recommended here.
- **Continuous integration**. Tools like Travis-CI automatically run a set of user-defined commands whenever changes are made to a version control repository. These commands typically execute tests to make sure that software hasn't regressed, i.e., that things which used to work still do. These tests can be run either before changes are saved (in which case, the changes can be rejected if something fails) or after (in which case, the project's contributors can be notified of the breakage). CI systems are invaluable in large projects with many contributors but pay fewer dividends in smaller projects where code is being written to do specific analyses.
- **Profiling and performance tuning**. Profiling is the act of measuring where a program spends its time and is an essential first step in tuning the program (i.e., making it run faster). Both are worth doing but only when the program's performance is actually a bottleneck; in our experience, most users spend more time getting the program right in the first place.
- **The semantic web**. Ontologies and other formal definitions of data are useful, but in our experience, even simplified things like Dublin Core are rarely encountered in the wild.
- **Documentation**. Good documentation is a key factor in software adoption, but in practice, people won't write comprehensive documentation until they have collaborators who will use it. They will, however, quickly see the point of a brief explanatory comment at the start of each script, so we have recommended that as a first step.
- **A bibliography manager**. Researchers should use a reference manager of some sort, such as Zotero, and should also obtain and use an ORCID to identify themselves in their publications, but discussion of those is outside the scope of this paper.
- **Code reviews and pair programming**. These practices are valuable in projects with multiple people making large software contributions, which is not typical for the intended audience for this paper [20].

One important observation about this list is that many experienced programmers actually do some or all of these things even for small projects. It makes sense for them to do so because (a) they've already paid the learning cost of the tool, so the time required to implement for the "next" project is small, and (b) they understand that their project will need some or all of these things as it scales up, so they might as well put it in place now.

The problem comes when those experienced developers give advice to people who haven't already mastered the tools and don't realize (yet) that they will save time if and when their project grows. In that situation, advocating unit testing with coverage checking and continuous integration is more likely to overwhelm newcomers rather than aid them.

## Conclusion

We have outlined a series of practices for scientific computing based on our collective experience and the experience of the thousands of researchers we have met through Software Carpentry, Data Carpentry, and similar organizations. These practices are pragmatic, accessible to people who consider themselves new to computing, and can be applied by both individuals and groups. Most importantly, these practices make researchers more productive individually by enabling them to get more done in less time and with less pain. They also accelerate research as a whole by making computational work (which increasingly means all work) more reproducible.

However, progress will not happen by itself. The practices described here are increasingly incentivized by requirements from journals and funding agencies, but the time and skills required to actually do them are still not being valued.

At a local level, principal investigators (PIs) can have the most impact, requiring that the research their lab produces follow these recommendations. Even if a PI doesn't have a background in computation, they can require that students show and share their code in lab meetings and with lab mates, those data are available and accessible to all in the lab, and that computational methods sections are comprehensive. PIs can also value the time it takes to do these things effectively and provide opportunities for training.

Universities can also support such efforts. While this is often provided by IT or high performance computing (HPC) groups, research librarians are an often underappreciated resource. Librarians have thought about and worked with data and provenance even before these computational challenges, and, increasingly, universities have dedicated data librarians on staff who have an explicit service role.

Many campuses also have self-organized groups led by students who wish to learn from each other, which may operate independently or in concert with organizations like Software Carpentry, Data Carpentry, or The Hacker Within.

Finally, many funding agencies now require data-management plans, education, and outreach activities. The true cost of implementing these plans includes training; it is unfair as well as counterproductive to insist that researchers do things without teaching them how. We believe it is now time for funders to invest in such training; we hope that our recommendations will help shape consensus on what "good enough" looks like and how to achieve it.
